# Deficiency of Inactive Rhomboid Protein 2 (iRhom2) Attenuates Macrophage Atherogenicity

**DOI:** 10.3390/biology15110860

**Published:** 2026-05-30

**Authors:** Carmen Hannemann, Alica Brettschneider, Phillip van Dijck, Karl Stangl, Antje Ludwig, Bernd Hewing

**Affiliations:** 1 Charité—Universitätsmedizin Berlin, Corporate Member of Freie Universität Berlin and Humboldt-Universität zu Berlin, Charitéplatz 1, 10117 Berlin, Germany; 2 Department of Cardiology, Angiology and Intensive Care Medicine, Deutsches Herzzentrum der Charité, Campus Charité Mitte, Charitéplatz 1, 10117 Berlin, Germany; 3 DZHK (German Centre for Cardiovascular Research), Partner Site Berlin, 10785 Berlin, Germany; 4 Zentrum für Kardiologie, Kardiologische Gemeinschaftspraxis, 48143 Muenster, Germany; 5 Department of Cardiology III—Adult Congenital and Valvular Heart Disease, University Hospital Muenster, 48149 Muenster, Germany

**Keywords:** iRhom2, TNF-α, monocytes, macrophages, atherosclerosis

## Abstract

Inactive rhomboid protein 2 (iRhom2) regulates ADAM17-mediated shedding of tumor necrosis factor-α (TNF-α) from immune cells. We previously showed that iRhom2 deficiency attenuates early atherosclerosis in mice. This study aimed to characterize the impact of iRhom2 deficiency on macrophage phenotype and function. Bone marrow-derived macrophages (BMDMs) from iRhom2^−/−^ and iRhom2^+/+^ mice were analyzed for proliferation, phagocytosis, survival of cytotoxic stress, and polarization. Cytokine secretion after LPS stimulation was quantified, and iRhom2 expression under atherogenic stimuli was assessed. Conditioned media from BMDMs (BMDMcM) were applied to human aortic endothelial cells (HAoECs) to evaluate adhesion molecule expression and monocyte adhesion. iRhom2 deficiency did not affect BMDM proliferation, phagocytosis, survival, or polarization marker expression. iRhom2 expression was upregulated in iRhom2^+/+^ BMDMs by atherogenic stimulation. Following LPS stimulation, TNF-α secretion was decreased and IL-10 secretion was increased in iRhom2^−/−^ compared with iRhom2^+/+^ BMDMs. HAoEC expression of adhesion molecules—ICAM-1, VCAM-1, and E-selectin—was attenuated after exposure to iRhom2^−/−^ compared with iRhom2^+/+^ BMDMcM. Monocyte adhesion to HAoECs was reduced following treatment with iRhom2^−/−^ BMDMcM; TNF-α neutralization abolished this effect, indicating TNF-α dependency. iRhom2 deficiency in BMDMs selectively alters macrophage inflammatory cytokine secretion without affecting basal macrophage functions, thereby reducing endothelial activation and monocyte adhesion. These findings identify iRhom2 as a regulator of macrophage–endothelial crosstalk and a potential target to modulate inflammation in atherogenesis.

## Simple Summary

Plaque buildup in blood vessels, called atherosclerosis, can lead to heart attacks and strokes. Inflammation from immune cells called macrophages plays a key role in starting this process. The protein iRhom2 helps macrophages to release an inflammatory molecule, TNF-alpha, which contributes to blood vessel inflammation. In the present study, macrophages without iRhom2 retained their normal functions—engulfing particles, surviving stress, and multiplying like regular cells—but released less TNF-alpha and more of the anti-inflammatory molecule IL-10. Messengers released from these macrophages caused blood vessel cells to produce fewer molecules that allow other immune cells to attach and spread inflammation. These findings suggest that iRhom2 plays a role in the early stages of blood vessel inflammation. Targeting iRhom2 could prevent or slow the initiation and progression of atherosclerosis without interfering with the normal function of immune cells.

## 1. Introduction

Atherosclerosis is a chronic inflammatory disease of the arterial vessel wall and a leading cause of death worldwide [1,2]. Its pathogenesis is characterized by the infiltration and accumulation of lipids and immune cells in the intimal arterial vessel wall. Macrophages are the dominant immune cell population within atherosclerotic lesions and critically determine plaque development and stability [1]. Beyond cytokine production, macrophage proliferation, apoptosis and efferocytosis are key processes in atherosclerotic plaque development. Local macrophage proliferation contributes substantially to lesional expansion in early disease, whereas increased apoptosis combined with impaired efferocytosis in advanced plaques promotes necrotic core formation and plaque destabilization [3,4].

Although macrophages exhibit a broad (heterogeneous) spectrum of characteristics, a conceptual model distinguishes between two opposing extremes: classically activated, proinflammatory M1 macrophages and alternatively activated, reparative M2 macrophages. M1 and M2 macrophages differ in receptor expression, cytokine/chemokine profile, and effector functions [5]. M1-like macrophages predominantly produce pro-inflammatory mediators such as tumor necrosis factor-α (TNF-α), interleukin (IL)-6, and IL-1β, whereas M2-like macrophages secrete anti-inflammatory cytokines and contribute to tissue repair and the resolution of inflammation. Progressing plaques are characterized by the enrichment of M1-like macrophages, whereas regressing lesions display a relative predominance of M2-like macrophages [6].

Endothelial activation and monocyte recruitment are early events in atherogenesis [7]. Circulating monocytes adhere to activated endothelial cells and transmigrate into the arterial intima, where they differentiate into macrophages and accumulate within the developing lesion. This process is mediated by adhesion molecules expressed on activated endothelial cells, including intercellular adhesion molecule-1 (ICAM-1), vascular cell adhesion molecule-1 (VCAM-1), and E-selectin, which facilitate firm monocyte adhesion and diapedesis [7,8].

TNF-α is a well-established activator of vascular endothelial cells [9]. It is primarily expressed by immune cells—particularly monocytes/macrophages (M1 phenotype) and T lymphocytes—as a transmembrane precursor [10]. Proteolytic cleavage by metalloprotease TNF-α converting enzyme (ADAM 17) releases the soluble, bioactive form of TNF-α [11]. Previous studies demonstrated that the activation and maturation of ADAM17 in hematopoietic cells requires inactive rhomboid protein 2 (iRhom2) [12,13]. Genetic deletion or silencing of iRhom2 prevents ADAM17 maturation and markedly reduces TNF-α shedding from immune cells, whereas ADAM17 function in non-hematopoietic cells remains largely preserved due to compensation by the related iRhom1 [14,15]. We previously demonstrated that iRhom2 deficiency attenuates early atherogenesis in mice [16]. To further elucidate the processes involved in early atherogenesis, the present study aimed to characterize the impact of iRhom2 deficiency on macrophage phenotype and function.

## 2. Materials and Methods

### 2.1. Cell Culture

Bone marrow-derived macrophages (BMDMs) were isolated and generated from bone marrow suspensions of the tibia and femur of 9- to 11-week-old male iRhom2^−/−^ mice and iRhom2^+/+^ littermate controls, as described previously [17]. iRhom2^−/−^ mice (kindly provided by Prof. Dr. Philipp Lang, Department of Molecular Medicine II, Universitätsklinikum Düsseldorf, Düsseldorf, Germany) and iRhom2^+/+^ littermate mice on a C57BL/6 genetic background were used for experiments. Genetic background control and breeding strategy have been described previously [16]. All animals were fed a standard diet. Bone marrow cells from 3–4 mice were pooled for each independent BMDM preparation and differentiated into BMDMs within seven days in RPMI-1640 medium (Thermo Fisher Scientific, Waltham, MA, USA) supplemented with 10% fetal bovine serum (Biochrom AG, Berlin, Germany), 1% penicillin/streptomycin (Thermo Fisher Scientific), and 15% L929 cell-conditioned RPMI medium at 37 °C in 5% CO_2_. Fresh medium was added or replaced every 2 to 3 days. All experiments were performed using a single batch of L929-conditioned medium that was prepared under standardized conditions, aliquoted, and stored at −80 °C. Successful macrophage differentiation with L929-conditioned medium was verified by cell morphology and by flow cytometric (CyAn ADP Analyzer; Beckman Coulter, Brea, CA, USA) analysis (Summit V4.3.02 software; Beckman Coulter) of established surface markers. At the end of BMDM differentiation, 95.47 ± 1.72% of iRhom2^+/+^ and 94.82 ± 1.42% of iRhom2^−/−^ BMDMs were positive for F4/80 (mouse F4/80-PE, clone Cl:A3-1; Abcam, Cambridge, UK). Among these F4/80-positive cells, >99% co-expressed CD11b (mouse CD11b-V450, clone M1/70; BD Biosciences, San Jose, CA, USA), with comparable expression levels between the two genotypes.

For classical and alternative activation, BMDMs were incubated with 1 µg/mL lipopolysaccharide (LPS; Sigma-Aldrich, St. Louis, MO, USA) or 20 ng/mL IL-4 (PromoCell, Heidelberg, Germany), respectively, for 6 h at 37 °C. To assess iRhom2 expression in macrophages in response to atherogenic stimuli, iRhom2^+/+^ BMDMs were stimulated with 50 ng/mL recombinant interferon-γ (IFN-γ; Biomol, Hamburg, Germany), 250 µM hydrogen peroxide (H_2_O_2_; Merck, Heidelberg, Germany), or 50 µg/mL oxidized low-density lipoprotein (oxLDL, Alfa Aesar, Waltham, MA, USA) for 6 h at 37 °C. iRhom2 mRNA expression was quantified by real-time RT-PCR.

For preparation of BMDM-conditioned medium (BMDMcM), mature iRhom2^−/−^ and iRhom2^+/+^ BMDMs (day 7) were stimulated with 100 ng/mL LPS in Endothelial Cell Growth Medium MV (ECGM, PromoCell) for 6 h at 37 °C (LPS-BMDMcM). Supernatants from untreated BMDMs were used as controls and are referred to as naïve BMDMcM.

For monocytes, cryopreserved THP-1 cells (ATCC, TIB-202) were cultured in RPMI supplemented with 10% fetal bovine serum and 1% penicillin/streptomycin. THP-1 cells were maintained at a density of 2–4 × 10^5^ cells/mL, as recommended by the vendor. The medium was refreshed or replaced as needed. THP-1 cells at passages P7 to P10 were used for experiments.

Human aortic endothelial cells (HAoECs; PromoCell, C-12271) were cultured in ECGM MV in T75 flasks. For passaging, cells were washed with Dulbecco’s phosphate-buffered saline (DPBS; Thermo Fisher Scientific) and detached using trypsin at 37 °C. Confluent HAoEC cultures were split in a 1:3 ratio onto new flasks. The medium was changed every other day. HAoECs at passages P5 to P7 were used for experiments.

### 2.2. Phagocytosis Assay

Differentiated BMDMs were harvested using Accutase (Sigma-Aldrich), seeded into 24-well plates (1 × 10^5^ cells/well), and cultured overnight. Phagocytic activity was determined using a commercial phagocytosis assay kit (Cayman Chemical, Ann Arbor, MI, USA; Cat. No. 500290) according to the manufacturer’s instructions. DAPI-stained nuclei and uptake of FITC-labeled beads were visualized by fluorescence microscopy (BIOREVO BZ 9000, Keyence, Osaka, Japan). For each genotype, five randomly selected fields per well were imaged with a 20× objective. Image analysis was performed with ImageJ software V1.48. Cells positive for both DAPI and FITC were counted as phagocytic cells, and total cell numbers were determined by DAPI-positive nuclei using the ImageJ cell counter tool. Phagocytosis was calculated as the percentage of phagocytic cells relative to the total cell number. Data were derived from *n* = 4 independent experiments.

### 2.3. Cell Viability and Cytotoxicity Assay

BMDMs were cultured in 12-well plates (2.4 × 10^6^ cells/well) for 7 days. Cell proliferation was assessed by measuring the number of viable cells at the start (0 h) and after 96 h. The MTT (3-(4,5-dimethylthiazol-2-yl)-2,5-diphenyltetrazoliumbromide; Sigma-Aldrich) assay was used to estimate the number of viable cells. Metabolically active cells convert MTT to dark blue, water-insoluble MTT–formazan. The formation of formazan was used as an indicator of the number of viable cells. Data were derived from *n* = 5 independent experiments. For cytotoxicity assay, BMDMs were rested in fresh medium for 1 h at 37 °C and then treated for 24 h with 0, 0.125, or 0.25 µM staurosporine (Sigma-Aldrich), or 0, 125, 250, 500, or 1000 µM H_2_O_2_ prior to MTT measurement. All measurements were performed in quadruplicate. Data were derived from *n* = 3–4 independent experiments.

### 2.4. Quantitative Real-Time RT-PCR

Cells were lysed in TRIzol reagent (Thermo Fisher Scientific) and 500 ng of total RNA was reverse-transcribed with random hexamers (TIB Molbiol, Berlin, Germany) using the Reverse Transcriptase Kit (Thermo Fisher Scientific). TaqMan Gene Expression Assays (Thermo Fisher Scientific) were used for the quantification of gene expression together with TaqMan Gene Expression Master Mix (Cat. No. 4369016). Specific assay IDs were as follows: *iRhom2* (Mm00553470_m1), *TNF-α* (Mm00443260_g1), *chemokine C-C motif ligand 2* (*CCL2*; Mm00441242_m1), *IL-1β* (Mm00434228_m1), *IL-6* (Mm00446190_m1), *inducible nitric oxide synthase* (*NOS2*; Mm00440502_m1), *arginase-1* (*Arg1*; Mm00475988_m1), *Fizz1* (*Retnla*; Mm00445109_m1), *mannose receptor* (*Mrc1*; Mm00485148_m1), and *IL-10* (Mm00439614_m1). *RPL19* (Mm02601633_g1) was used as a housekeeping gene. qPCR was performed using the 7300 Real Time PCR System (Applied Biosystems, Waltham, MA, USA). Relative gene expression was calculated using the 2^−ΔΔCt^ method, with Ct values normalized to the housekeeping gene and expressed relative to the control group. Data were derived from *n* = 3–4 independent experiments. Raw Ct values for RPL19 were stable across all experimental conditions, with no observable variance between treatment and control groups or between genotypes.

### 2.5. Measurements of Inflammatory Mediators

Levels of TNF-α, IFN-γ, IFN-β, IL-6, IL-1α, IL-1β, IL12p70, IL-17A, IL-23, IL-27, CCL2, IL-10 and granulocyte-macrophage colony-stimulating factor (GM-CSF) were measured in LPS-BMDMcM using a bead-based immunoassay (LEGENDplex™, BioLegend, San Diego, CA, USA; Cat. No. 740446) according to the manufacturer’s instructions. Data were derived from *n* = 3 independent experiments. IL-10 and TNF-α were additionally measured by ELISA in the supernatant of BMDMs stimulated with LPS (1 µg/mL). IL-10 was measured using the Mouse IL-10 ELISA MAX™ Deluxe Set (BioLegend), and TNF-α was measured using the BD OptEIA™ Mouse TNF ELISA Set II (BD Bioscience) with BD OptEIA™Reagent Set A (BD Bioscience), following the manufacturer’s protocol. Data were derived from *n* = 3 independent BMDM preparations.

### 2.6. Cell-Based ELISA for Adhesion Molecule Expression

HAoECs were seeded in 96-well plates (1 × 10^4^ cells/well) and cultured for 3 days until confluence. Medium was replaced one day before stimulation. HAoECs were stimulated with naïve or LPS BMDMcM (1:4 dilution in ECGM) for 4 h at 37 °C. Based on initial assay optimization, a 1:4 dilution of BMDMcM in fresh ECGM was utilized to ensure HAoEC viability and prevent direct activation by residual LPS. Cells incubated with ECGM alone served as controls. Each condition was performed in triplicate. Following stimulation, cells were fixed with 0.2% glutaraldehyde (Sigma-Aldrich) for 10 min at room temperature and washed with wash buffer (0.05% BSA, 0.02% Triton X-100 in DPBS). Cells were incubated with primary antibodies against human ICAM-1 (clone 84H10; Beckman Coulter), VCAM-1 (clone 1G11; Beckman Coulter), or E-selectin (clone 1.2B6; Sigma-Aldrich) diluted 1:500 in antibody buffer for 1 h. After washing, cells were incubated with biotinylated rabbit anti-mouse IgG (1:500; Jackson ImmunoResearch, West Grove, PA, USA) for 45 min, followed by streptavidin–HRP complex (1:500; GE HealthCare, Freiburg, Germany) for 45 min. After washing, OPD substrate solution (Dako, Hamburg, Germany) prepared in citrate buffer containing 0.05% H_2_O_2_ was added and incubated for 5–10 min, protected from light. The reaction was stopped with 3 M sulfuric acid (H_2_SO_4_), and absorbance was measured at 492 nm (reference 620 nm) using a SpectraMax 340PC384 microplate reader (Molecular Devices, San Jose, CA, USA). All incubation and washing steps were performed at room temperature with gentle shaking. Data are derived from *n* = 4 independent BMDM-conditioned media preparations.

### 2.7. Monocyte Adhesion Assay

Monocyte adhesion was assessed by activating HAoECs with naïve and LPS-BMDMcM for 4 h at 37 °C, as described above. Following incubation, conditioned medium was removed and HAoECs were washed twice with prewarmed DPBS before adding Calcein AM (Thermo Fisher Scientific)-labeled THP-1 monocytes for 30 min. Non-adherent cells were removed by washing three times with prewarmed DPBS, and the fluorescence of adherent monocytes was quantified using a fluorescence plate reader GEMINI EM Microplate Reader (Molecular Devices). For TNF-α neutralization, LPS-BMDMcM was preincubated with an anti-mouse TNF-α antibody (clone D2H4; Cell Signaling Technology, Danvers, MA, USA) or an isotype-matched IgG control (clone DA1E; Cell Signaling Technology) for 1 h at 37 °C before addition to HAoECs. Monocyte adhesion was then assessed as described above. Data are derived from *n* = 4–5 independent BMDM-conditioned media preparations.

### 2.8. Statistical Analysis

Data were analyzed using GraphPad Prism Software (version 10.2.3). Sample sizes (*n* = 3–5) represent independent biological replicates. Unless otherwise indicated, summary data are presented as the mean ± standard error of the mean (SEM) with individual data points plotted to display distribution. The normality of data was assessed using the Shapiro–Wilk test. Comparisons between two independent groups were made using an unpaired *t*-test with Welch’s correction for normally distributed data. Datasets consistent with lognormal distributions were log-transformed prior to statistical testing to satisfy parametric assumptions. For experiments involving multiple independent variables across genotypes, two-way analysis of variance (ANOVA) followed by Tukey’s or Sidak’s multiple-comparisons test was used, as indicated; *p* < 0.05 was considered statistically significant.

## 3. Results

### 3.1. Impact of iRhom2 Deficiency on Macrophage Phagocytosis, Viability, Survival, and Polarization

**Figure 1:**
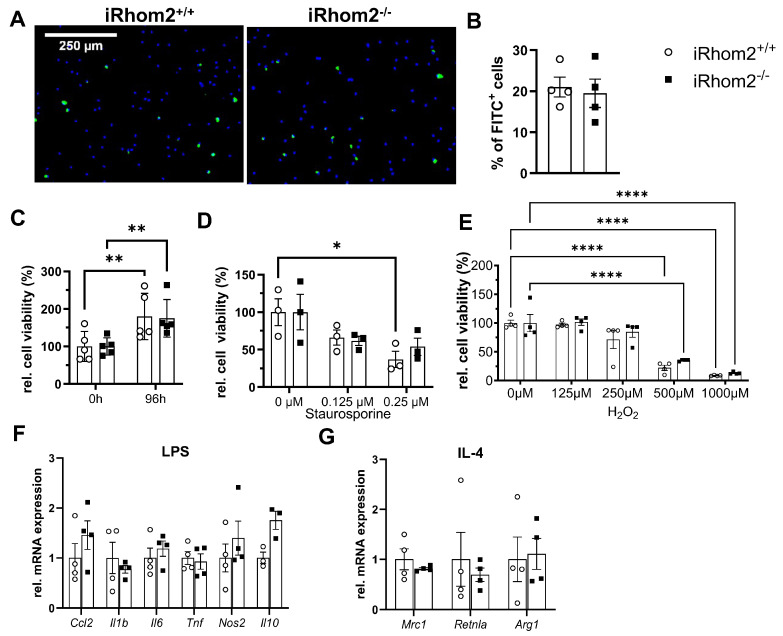
**iRhom2 deficiency does not alter the phagocytosis capacity, proliferation, or susceptibility to cytotoxic stress of bone marrow-derived macrophages under basal conditions and does not impair their polarization toward M1 and M2 phenotypes.** (**A**) Representative images of bone marrow-derived macrophages (BMDMs) from iRhom2^+/+^ and iRhom2^−/−^ mice after 1 h incubation with FITC-labeled beads. Cell nuclei were visualized using DAPI (4′,6-diamidino-2-phenylindole); scale bar: 250 µm. (**B**) Quantification of phagocytosed beads relative to total cell number (DAPI+ cells). *n* = 4 independent experiments. (**C**–**E**) Cell viability of iRhom2^+/+^ and iRhom2^−/−^ BMDMs was assessed by MTT assay. (**C**) Proliferation was assessed by measuring metabolic activity (OD at 570 nm) at 0 h and 96 h of culture. OD at 0 h was set to 100%. *n* = 5 independent experiments. (**D**,**E**) Survival was estimated by measuring cell viability after treatment with the indicated concentration of staurosporine or hydrogen peroxide (H_2_O_2_) for 24 h. Optical density (OD) at 570 nm of untreated cells (0 µm staurosporine or H_2_O_2_) was set to 100%. *n* = 3–4 independent experiments. (**F**,**G**) mRNA expression was analyzed by real-time PCR after 6 h stimulation of BMDMs with lipopolysaccharide (LPS, classical activation) or interleukin-4 (IL-4, alternative activation). Gene expression of inflammatory markers (chemokine (C-C motif) ligand 2, *Ccl2*; interleukin (IL)-1β, *Il1β*; IL-6, *Il6*; inducible nitric oxide synthase, *Nos2*; tumor necrosis factor-α, *Tnf*), immunomodulatory marker IL-10 (*Il10*), and markers of alternative activation (mannose receptor, *Mrc1*; Fizz1, *Retnla*; arginase-1, *Arg1*). *n* = 3–4 independent experiments. Data are presented as mean ± SEM with individual data points. * *p* < 0.05, ** *p* < 0.01, **** *p* < 0.0001 versus timepoint or treatment (as indicated), determined by two-way ANOVA followed by Sidak’s multiple-comparisons test. iRhom2, inactive rhomboid protein 2.

Phagocytosis, proliferation, apoptosis, and polarization are fundamental macrophage functions that critically determine atherosclerotic lesion development [4]. Phagocytosis capacity, measured by the uptake of FITC-labeled beads within one hour, did not differ significantly between iRhom2^+/+^ and iRhom2^−/−^ BMDMs (Figure 1A,B). To assess proliferation, we used the MTT assay to measure metabolic activity as a surrogate for viable cell accumulation. Over 96 h of culture, both genotypes exhibited a comparable increase in metabolically active cell mass, with no significant differences between iRhom2^+/+^ and iRhom2^−/−^ BMDMs, indicating a similar proliferation rate (Figure 1C). Regarding susceptibility to apoptotic stress, the treatment of BMDMs with 0.125 and 0.25 µM staurosporine, a known inducer of apoptosis, reduced cell viability in both iRhom2^+/+^ and iRhom2^−/−^ BMDMs compared to untreated controls; however, this reduction reached statistical significance only with 0.25 µM staurosporine in iRhom2^+/+^ BMDMs (Figure 1D). Despite this difference, the overall susceptibility to staurosporine-induced cytotoxicity was comparable between the two genotypes. Similarly, H_2_O_2_ treatment reduced cell viability in both genotypes, with no differences observed between iRhom2^+/+^ and iRhom2^−/−^ BMDMs (Figure 1E). To determine whether iRhom2 deficiency affects macrophage polarization, iRhom2^+/+^ and iRhom2^−/−^ BMDMs were stimulated with LPS or IL-4 to induce classical or alternative activation, respectively, followed by quantitative real-time PCR analysis. Gene expression of inflammatory markers (*Ccl2*, *Il6*, *Il1b*, *Nos2*, *Tnf*; Figure 1F), the immunomodulatory marker *Il10* (Figure 1F), and alternative activation markers (*Arg1*, *Mrc1*, *Retnla*; Figure 1G) was not significantly different between iRhom2^+/+^ and iRhom2^−/−^ BMDMs. Taken together, these data show that basal macrophage functions, including phagocytosis, proliferation, survival under cytotoxic stress, and polarization, are preserved in the absence of iRhom2.

### 3.2. iRhom2 Expression in Macrophages Is Upregulated by Atherogenic Stimuli

**Figure 2:**
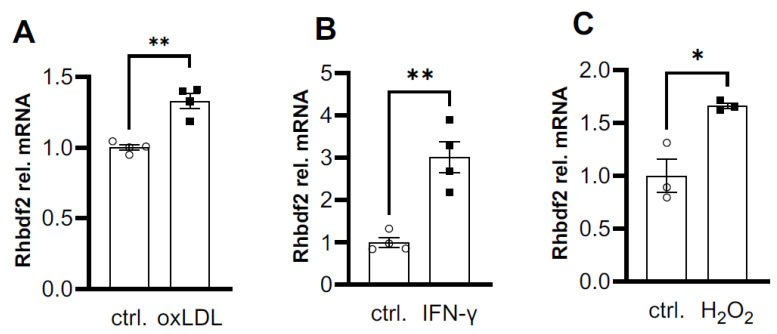
**iRhom2 expression in bone marrow-derived macrophages is upregulated by atherogenic stimuli.** iRhom2 mRNA expression was quantified by real-time RT-PCR in iRhom2^+/+^ bone marrow-derived macrophages (BMDMs) stimulated with (**A**) oxidized low-density lipoprotein (oxLDL; 50 µg/mL), (**B**) interferon-γ (IFN-γ; 50 ng/mL), (**C**) hydrogen peroxide (H_2_O_2_; 250 µM), or corresponding vehicle control (ctrl.) for 6 h. *n* = 3–4 independent experiments. Data are presented as mean ± SEM with individual data points. * *p* < 0.05, ** *p* < 0.01 versus vehicle control, determined by Welch’s *t*-test. dots: vehicle control, black square: stimulation.

iRhom2 mRNA expression in macrophages increases in response to LPS stimulation [12]. To determine whether atherogenic stimuli similarly regulate iRhom2, iRhom2^+/+^ BMDMs were treated with oxidized LDL (oxLDL), IFN-γ, or H_2_O_2_ (Figure 2A–C). All three stimuli significantly upregulated iRhom2 mRNA expression in macrophages. Taken together, these data indicate that iRhom2 is responsive to atherogenic stimuli, suggesting a potential role in macrophage activation under atherogenic conditions.

### 3.3. Impact of iRhom2 Deficiency on Inflammatory Mediator Secretion by Macrophages

**Figure 3:**
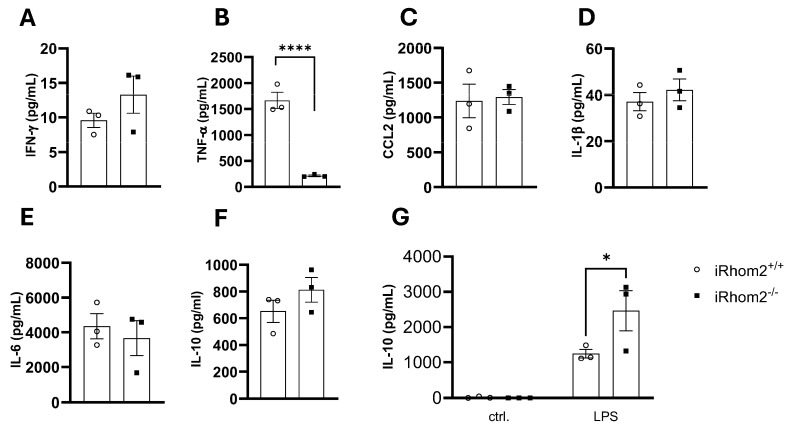
**Secretion of inflammatory mediators by lipopolysaccharide-stimulated bone marrow-derived macrophages.** (**A**–**E**) Bone marrow-derived macrophages (BMDMs) from iRhom2^+/+^ and iRhom2^−/−^ mice were stimulated with lipopolysaccharide (LPS) for 6 h, and supernatants were analyzed using a bead-based immunoassay. Shown are levels of (**A**) interferon-γ (IFN-γ); (**B**) tumor necrosis factor-α (TNF-α); (**C**) C-C motif ligand 2 (CCL2); (**D**) interleukin-1beta (IL-1β); (**E**) IL-6; (**F**) IL-10. *n* = 3 independent experiments. Data are presented as mean ± SEM with individual data points. **** *p* < 0.0001 versus genotype (as indicated), determined by Welch’s *t*-test. (**G**), BMDMs from iRhom2^+/+^ and iRhom2^−/−^ mice were stimulated with LPS or vehicle control (ctrl.) for 24 h. IL-10 levels in the supernatant were assessed by enzyme-linked immunosorbent assay (ELISA). *n* = 3 independent experiments. Data are presented as mean ± SEM with individual data points. * *p* < 0.05 versus genotype and treatment (as indicated), determined by two-way ANOVA followed by Sidak’s multiple-comparisons test. iRhom2, inactive rhomboid protein 2.

Pro-inflammatory macrophages are enriched in progressing atherosclerotic plaques [6]. To assess the impact of iRhom2 deficiency on inflammatory mediator secretion, supernatants from LPS-stimulated iRhom2^+/+^ and iRhom2^−/−^ BMDMs were analyzed. Compared with supernatants from iRhom2^+/+^ BMDMs, TNF-α protein levels were significantly lower in the iRhom2^−/−^ BMDM supernatants (iRhom2^+/+^: 1667 ± 271 pg/mL vs. iRhom2^−/−^: 213 ± 26 pg/mL, *p* < 0.05); in contrast, protein levels of IFN-γ, IL-6, IL-1α, IL-1β, IL-17A, CCL2, IFN-β, IL-23, IL-27, IL-12p70, GM-CSF, and IL-10 in the supernatant did not differ significantly between both genotypes after 6 h of LPS stimulation of BMDMs (Figure 3A–F and Supplementary Figure S1A–G). Because IL-10 secretion typically occurs at later timepoints following LPS stimulation, IL-10 levels were measured by ELISA after 24 h of LPS stimulation of BMDMs. At this timepoint, IL-10 protein levels were significantly higher in supernatants of iRhom2^−/−^ BMDMs compared with iRhom2^+/+^ BMDMs (*p* < 0.05) (Figure 3G). In contrast, TNF-α levels had declined in iRhom2^+/+^ BMDMs and returned to near-baseline in iRhom2^−/−^ BMDMs at 24 h (Supplementary Figure S1H). Taken together, iRhom2 deficiency in macrophages alters TNF-α and IL-10 secretion, consistent with a shift toward a less inflammatory macrophage phenotype.

### 3.4. Impact of iRhom2 Deficiency on Monocyte Adhesion

**Figure 4:**
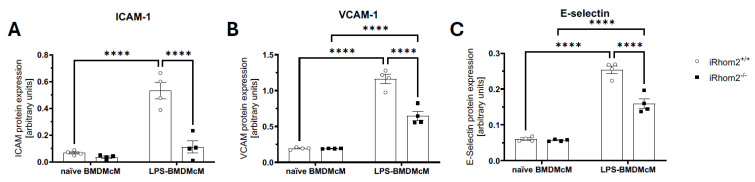
**iRhom2 deficiency reduces endothelial adhesion molecule expression induced by conditioned medium from lipopolysaccharide-stimulated bone marrow-derived macrophages.** Human aortic endothelial cells (HAoECs) were treated for 4 h with conditioned medium from bone marrow-derived macrophages (BMDMcM) from iRhom2^+/+^ and iRhom2^−/−^ mice cultured under basal conditions (naïve) or stimulated with lipopolysaccharide (LPS). Expression of (**A**) intracellular adhesion molecule-1 (ICAM-1), (**B**) vascular cell adhesion molecule-1 (VCAM-1), and (**C**) E-selectin (also known as endothelial-leukocyte adhesion molecule-1, ELAM-1) was assessed by cell-based enzyme-linked immunosorbent assay (ELISA). *n* = 4 independent experiments. Data are presented as mean ± SEM with individual data points. **** *p* < 0.0001 versus genotype or treatment (as indicated), determined by two-way ANOVA followed by Tukey’s multiple-comparisons test. iRhom2, inactive rhomboid protein 2.

Monocyte adhesion to vascular endothelial cells is a key step in atherogenesis, contributing to lesion initiation and progression, with TNF-α serving as one of the central activators of endothelial cell adhesion molecule expression [18]. To assess the impact of macrophage iRhom2 on endothelial activation, HAoECs were treated with conditioned medium from either naïve (naïve BMDMcM) or LPS-stimulated (LPS-BMDMcM) iRhom2^+/+^ or iRhom2^−/−^ BMDMs. Treatment with naïve BMDMcM of either genotype did not significantly alter ICAM-1, VCAM-1, and E-selectin expression on HAoECs (Figure 4A–C). LPS-BMDMcM from iRhom2^+/+^ BMDMs significantly upregulated all three adhesion molecules compared with naïve BMDMcM. In contrast, LPS-BMDMcM from iRhom2^−/−^ BMDMs did not significantly increase ICAM-1 expression (Figure 4A) and produced only attenuated increases in VCAM-1 and E-selectin expression (Figure 4B,C). Consequently, HAoEC expression of ICAM-1, VCAM-1, and E-selectin was significantly lower following exposure to LPS-BMDMcM from iRhom2^−/−^ compared with iRhom2^+/+^ BMDMs.

**Figure 5:**
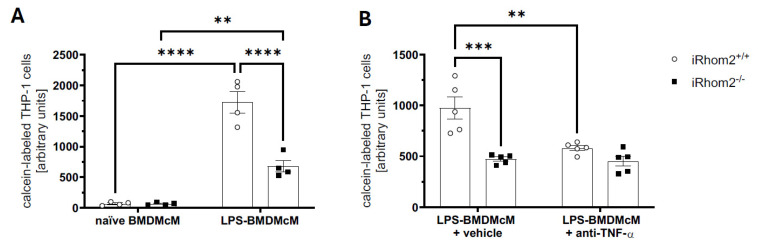
**Lipopolysaccharide-conditioned medium from iRhom2-deficient bone marrow-derived macrophages reduces monocyte adhesion to endothelial cells in a TNF-α-dependent manner.** Bone marrow-derived macrophages (BMDMs) from iRhom2^+/+^ and iRhom2^−/−^ mice were cultured for 6 h in the absence or presence of lipopolysaccharide (LPS) to generate naïve or LPS-conditioned macrophage medium (BMDMcM). (**A**) Human aortic endothelial cells (HAoECs) were simulated for 4 h with naïve or LPS BMDMcM, and the adhesion of calcein-labeled THP-1 monocytes was measured using a fluorescence plate reader. *n* = 4 independent experiments. (**B**) LPS BMDMcM was pretreated with a tumor necrosis factor-α (TNF-α)-neutralizing antibody (anti-TNF-α) or isotype control (vehicle) before performing the monocyte adhesion assay described in (**A**). *n* = 5 independent experiments. Data are presented as mean ± SEM with individual data points. ** *p* < 0.01, *** *p* < 0.001, **** *p* < 0.0001 versus genotype or treatment (as indicated), determined by two-way ANOVA followed by Tukey’s multiple-comparisons test. iRhom2, inactive rhomboid protein 2.

Activation of HAoECs with LPS-BMDMcM from both genotypes significantly increased THP-1 monocyte adhesion compared with naïve BMDMcM. However, adhesion induced by LPS-BMDMcM from iRhom2^−/−^ BMDMs was ~60% lower than that induced by LPS-BMDMcM of iRhom2^+/+^ BMDMs (Figure 5A).

Neutralization of TNF-α in LPS-BMDMcM from iRhom2^+/+^ BMDMs significantly reduced monocyte adhesion compared with non-neutralized LPS-BMDMcM and almost abolished the differences between both genotypes (Figure 5B).

Taken together, these results indicate that reduced soluble TNF-α secretion by iRhom2-deficient macrophages attenuates endothelial cell activation and subsequent monocyte adhesion.

## 4. Discussion

In the present study, we provide mechanistic insight into how iRhom2 modulates macrophage-driven inflammation. Our data demonstrate that iRhom2 deficiency does not affect fundamental macrophage characteristics, including survival, phagocytosis, proliferation, or M1- and M2-like polarization—processes that are critical for resident intimal macrophage function and recruited monocyte-derived macrophages throughout atherogenesis.

Macrophage apoptosis is the predominant form of cell death in atherosclerotic plaques, particularly in advanced lesions [3]. The unchanged survival rate of iRhom2-deficient macrophages in response to cytotoxic stress indicates that iRhom2 does not regulate macrophage survival or turnover. Efficient efferocytosis, the phagocytic clearance of apoptotic cells by neighboring macrophages, is essential in early lesions to maintain tissue homeostasis and limit inflammation. In contrast, impaired efferocytosis in advanced plaques promotes the accumulation of apoptotic debris and secondary necrosis, driving plaque progression and instability [19]. Notably, iRhom2 deficiency did not affect the fundamental machinery for phagocytosis (as evidenced by the uptake of FITC-labeled beads), indicating that efferocytosis-related functions remain intact. Whether iRhom2 specifically modulates the complex signaling pathways involved in efferocytosis within the necrotic core of atherosclerotic plaques needs to be elucidated in future studies.

Local macrophage proliferation contributes to the expansion of the lesional macrophage pool [4]. Because proliferative capacity was preserved in iRhom2-deficient macrophages, the previously observed atheroprotective phenotype in iRhom2-deficient mice [16] is unlikely attributable to reduced macrophage accumulation through local proliferation. Taken together, these findings indicate that iRhom2 does not influence macrophage survival, efferocytosis, or proliferation, supporting the conclusion that its atheroprotective effects are mediated primarily through modulation of inflammatory signaling rather than quantitative changes in macrophage populations.

iRhom2 deficiency also did not affect macrophage polarization: mRNA expression of typical M1/M2 markers remained unchanged following LPS or IL-4 stimulation. This suggests that iRhom2 does not regulate transcriptional polarization programs, further supporting the notion that its impact is independent of macrophage differentiation. In addition, these findings are consistent with Barnette et al. [20], showing that iRhom2 deficiency does comprise macrophage responsiveness to pro-inflammatory or anti-inflammatory stimuli. However, while Barnette et al. reported impaired macrophage differentiation and phagocytosis in iRhom2-deficient bone marrow-derived macrophages, we did not observe such alterations under our experimental conditions. These differences may reflect context-dependent effects of iRhom2 on macrophage function, potentially influenced by genetic background (C57BL/6 vs. 129/SV) and experimental design, including the use of non-littermate wild-type controls in their study [21].

While macrophage basal functions are preserved in the present study, the altered inflammatory phenotype of iRhom2-deficient macrophages arises from changes in cytokine shedding. Despite an intact macrophage polarization, iRhom2-deficient macrophages displayed a markedly altered secretory pattern, characterized by reduced TNF-α and increased IL-10 release. These observations indicate that iRhom2 deficiency does not impair macrophage activation, per se, but selectively alters the inflammatory secretome, thereby limiting the propagation of inflammation to recipient cells. TNF-α is known to induce IL-10 expression via the activation of its receptors, forming a self-regulating negative feedback loop in which IL-10 subsequently suppresses TNF-α secretion [22,23]. Activation of TNF-receptor-2 (TNFR2) appears to preferentially induce IL-10 production [24,25]. In iRhom2-deficient macrophages, impaired ADAM17-mediated cleavage increases the surface expression of tmTNF-α [13]. Because tmTNF-α preferentially signals through TNFR2 rather than TNFR1, enhanced TNFR2 engagement may mechanistically explain the observed increase in IL-10 expression and secretion [26]. IL-10 exerts well-established atheroprotective effects, including the reduction in atherosclerotic lesion size and the promotion of favorable plaque composition in experimental models; in humans, IL-10 levels have been associated with lower atherosclerotic plaque burden [27,28,29].

Functionally, this altered macrophage secretome exhibited a reduced capacity to induce endothelial activation, as evidenced by decreased expression of ICAM-1, VCAM-1, and E-selectin. Because endothelial expression of these adhesion molecules is critical for leukocyte recruitment [7], this resulted in decreased monocyte adhesion. Importantly, this effect was TNF-α-dependent, highlighting the central role of iRhom2-regulated TNF-α shedding in macrophage–endothelial crosstalk.

We further observed that iRhom2 expression in macrophages is induced by multiple atherogenic stimuli, including IFN-γ, oxidized LDL, and hydrogen peroxide, all of which are abundant within the atherosclerotic plaque microenvironment [30,31]. This inducibility underscores the dynamic regulation of iRhom2 under proatherogenic conditions and its pathophysiological relevance in atherosclerosis.

Targeting inflammation in atherosclerosis has emerged as a therapeutic strategy to reduce cardiovascular risk beyond lipid lowering [32,33]. Our findings suggest that iRhom2 may represent a target to selectively modulate immune-driven inflammation from early stages of disease. Importantly, this approach preserves essential macrophage functions—proliferation, phagocytosis, survival, and polarization—while limiting pathological TNF-α-driven responses. Moreover, ADAM17-dependent processes in non-immune cells remain largely intact due to compensatory iRhom1 activity [14], highlighting the potential for immune cell-specific intervention. By selectively dampening macrophage pro-inflammatory activity without broadly suppressing systemic immunity, iRhom2-targeted therapies may provide a precision strategy to limit atherogenesis while minimizing adverse effects associated with global TNF-α inhibition [15,34].

The limitations of our study include the in vitro nature of mechanistic assays, which may not fully capture the complexity of in vivo plaque microenvironments and may restrict the translational relevance of the findings. Furthermore, the relatively limited number of experimental replicates should be considered when interpreting the results. In addition, the use of an early 6 h timepoint for macrophage polarization may not capture the maximal induction of M2 markers with later induction kinetics, such as *Arg1* and *RetnIa*. Future studies are warranted to further define the role of iRhom2 in macrophage–endothelial crosstalk and efferocytosis under complex inflammatory in vivo conditions. Macrophage-specific targeting of iRhom2 should be explored as a potential strategy to modulate vascular inflammation in a more selective manner. Further investigation is also required to determine whether iRhom2-targeted interventions exert comparable anti-inflammatory effects in human macrophages and to perform unbiased profiling of the iRhom2-regulated macrophage secretome, including the contribution of additional ADAM17 substrates.

## 5. Conclusions

Our data demonstrate that iRhom2 deficiency in bone marrow-derived macrophages selectively modulates inflammatory signaling without affecting core macrophage functions, resulting in reduced endothelial activation and monocyte recruitment in our experimental setting. These findings indicate a role for iRhom2 in macrophage-driven inflammatory responses in atherogenesis and provide a mechanistic basis for targeting iRhom2 to modulate vascular inflammation.

## Data Availability

Data will be made available on request.

## Supplementary Materials

The following supporting information can be downloaded at: https://www.mdpi.com/article/10.3390/biology15110860/s1, Supplementary Figure S1. Secretion of inflammatory mediators by lipopolysaccharide-stimulated bone marrow-derived macrophages.

## References

[1] Libby P. (2012). Inflammation in Atherosclerosis. Arterioscler. Thromb. Vasc. Biol..

[2] Roth G.A., Mensah G.A., Johnson C.O., Addolorato G., Ammirati E., Baddour L.M., Barengo N.C., Beaton A.Z., Benjamin E.J., Benziger C.P. (2020). Global Burden of Cardiovascular Diseases and Risk Factors, 1990–2019. J. Am. Coll. Cardiol..

[3] Tabas I., Bornfeldt K.E. (2016). Macrophage Phenotype and Function in Different Stages of Atherosclerosis. Circ. Res..

[4] Hou P., Fang J., Liu Z., Shi Y., Agostini M., Bernassola F., Bove P., Candi E., Rovella V., Sica G. (2023). Macrophage Polarization and Metabolism in Atherosclerosis. Cell Death Dis..

[5] Murray P.J., Allen J.E., Biswas S.K., Fisher E.A., Gilroy D.W., Goerdt S., Gordon S., Hamilton J.A., Ivashkiv L.B., Lawrence T. (2014). Macrophage Activation and Polarization: Nomenclature and Experimental Guidelines. Immunity.

[6] Barrett T.J. (2020). Macrophages in Atherosclerosis Regression. Arterioscler. Thromb. Vasc. Biol..

[7] Blankenberg S., Barbaux S., Tiret L. (2003). Adhesion Molecules and Atherosclerosis. Atherosclerosis.

[8] Pickett J.R., Wu Y., Zacchi L.F., Ta H.T. (2023). Targeting Endothelial Vascular Cell Adhesion Molecule-1 in Atherosclerosis: Drug Discovery and Development of Vascular Cell Adhesion Molecule-1–Directed Novel Therapeutics. Cardiovasc. Res..

[9] Bradley J. (2008). TNF—Mediated Inflammatory Disease. J. Pathol..

[10] Van Loo G., Bertrand M.J.M. (2023). Death by TNF: A Road to Inflammation. Nat. Rev. Immunol..

[11] Black R.A., Rauch C.T., Kozlosky C.J., Peschon J.J., Slack J.L., Wolfson M.F., Castner B.J., Stocking K.L., Reddy P., Srinivasan S. (1997). A Metalloproteinase Disintegrin That Releases Tumour-Necrosis Factor-α from Cells. Nature.

[12] McIlwain D.R., Lang P.A., Maretzky T., Hamada K., Ohishi K., Maney S.K., Berger T., Murthy A., Duncan G., Xu H.C. (2012). iRhom2 Regulation of TACE Controls TNF-Mediated Protection Against *Listeria* and Responses to LPS. Science.

[13] Adrain C., Zettl M., Christova Y., Taylor N., Freeman M. (2012). Tumor Necrosis Factor Signaling Requires iRhom2 to Promote Trafficking and Activation of TACE. Science.

[14] Issuree P.D.A., Maretzky T., McIlwain D.R., Monette S., Qing X., Lang P.A., Swendeman S.L., Park-Min K.-H., Binder N., Kalliolias G.D. (2013). iRHOM2 Is a Critical Pathogenic Mediator of Inflammatory Arthritis. J. Clin. Investig..

[15] Calligaris M., Cuffaro D., Bonelli S., Spanò D.P., Rossello A., Nuti E., Scilabra S.D. (2021). Strategies to Target ADAM17 in Disease: From Its Discovery to the iRhom Revolution. Molecules.

[16] Hannemann C., Schecker J.H., Brettschneider A., Grune J., Rösener N., Weller A., Stangl V., Fisher E.A., Stangl K., Ludwig A. (2022). Deficiency of Inactive Rhomboid Protein 2 (iRhom2) Attenuates Diet-Induced Hyperlipidaemia and Early Atherogenesis. Cardiovasc. Res..

[17] Zhang X., Goncalves R., Mosser D.M. (2008). The Isolation and Characterization of Murine Macrophages. Curr. Protoc. Immunol..

[18] Ait-Oufella H., Taleb S., Mallat Z., Tedgui A. (2011). Recent Advances on the Role of Cytokines in Atherosclerosis. Arterioscler. Thromb. Vasc. Biol..

[19] Tabas I. (2010). Macrophage Death and Defective Inflammation Resolution in Atherosclerosis. Nat. Rev. Immunol..

[20] Barnette D.N., Cahill T.J., Gunadasa-Rohling M., Carr C.A., Freeman M., Riley P.R. (2018). iRhom2-Mediated Proinflammatory Signalling Regulates Heart Repair Following Myocardial Infarction. JCI Insight.

[21] Doetschman T. (2009). Influence of Genetic Background on Genetically Engineered Mouse Phenotypes. Methods Mol. Biol..

[22] Daftarian P.M., Kumar A., Kryworuchko M., Diaz-Mitoma F. (1996). IL-10 Production Is Enhanced in Human T Cells by IL-12 and IL-6 and in Monocytes by Tumor Necrosis Factor-Alpha. J. Immunol..

[23] Saraiva M., O’Garra A. (2010). The Regulation of IL-10 Production by Immune Cells. Nat. Rev. Immunol..

[24] Gane J.M., Stockley R.A., Sapey E. (2016). TNF-α Autocrine Feedback Loops in Human Monocytes: The Pro- and Anti-Inflammatory Roles of the TNF-α Receptors Support the Concept of Selective TNFR1 Blockade In Vivo. J. Immunol. Res..

[25] Veroni C., Gabriele L., Canini I., Castiello L., Coccia E., Remoli M.E., Columba-Cabezas S., Aricò E., Aloisi F., Agresti C. (2010). Activation of TNF Receptor 2 in Microglia Promotes Induction of Anti-Inflammatory Pathways. Mol. Cell. Neurosci..

[26] Grell M., Douni E., Wajant H., Löhden M., Clauss M., Maxeiner B., Georgopoulos S., Lesslauer W., Kollias G., Pfizenmaier K. (1995). The Transmembrane Form of Tumor Necrosis Factor Is the Prime Activating Ligand of the 80 kDa Tumor Necrosis Factor Receptor. Cell.

[27] Fourman L.T., Saylor C.F., Cheru L., Fitch K., Looby S., Keller K., Robinson J.A., Hoffmann U., Lu M.T., Burdo T. (2020). Anti-Inflammatory Interleukin 10 Inversely Relates to Coronary Atherosclerosis in Persons With Human Immunodeficiency Virus. J. Infect. Dis..

[28] Mallat Z., Besnard S., Duriez M., Deleuze V., Emmanuel F., Bureau M.F., Soubrier F., Esposito B., Duez H., Fievet C. (1999). Protective Role of Interleukin-10 in Atherosclerosis. Circ. Res..

[29] Kleemann R., Zadelaar S., Kooistra T. (2008). Cytokines and Atherosclerosis: A Comprehensive Review of Studies in Mice. Cardiovasc. Res..

[30] Voloshyna I., Littlefield M.J., Reiss A.B. (2014). Atherosclerosis and Interferon-γ: New Insights and Therapeutic Targets. Trends Cardiovasc. Med..

[31] Madamanchi N.R., Vendrov A., Runge M.S. (2005). Oxidative Stress and Vascular Disease. Arterioscler. Thromb. Vasc. Biol..

[32] Ridker P.M., Everett B.M., Thuren T., MacFadyen J.G., Chang W.H., Ballantyne C., Fonseca F., Nicolau J., Koenig W., Anker S.D. (2017). Antiinflammatory Therapy with Canakinumab for Atherosclerotic Disease. N. Engl. J. Med..

[33] Soehnlein O., Libby P. (2021). Targeting Inflammation in Atherosclerosis—From Experimental Insights to the Clinic. Nat. Rev. Drug Discov..

[34] Atzeni F., Nucera V., Gerratana E., Cirillo M., Marino F., Miceli G., Sangari D., Boccassini L., Masala I.F. (2020). Concerns about the Safety of Anti-TNF Agents When Treating Rheumatic Diseases. Expert Opin. Drug Saf..

