# Supplementary material for: Deficiency of Inactive Rhomboid Protein 2 (iRhom2) Attenuates Macrophage Atherogenicity
Source: Biology (Basel). 2026 May 30;15(11):860. doi: 10.3390/biology15110860 (PMC13255711; doi:10.3390/biology15110860)
Supplement: Supplementary file 1 [file biology-15-00860-s001.zip › biology-4297589-supplementary.pdf]

Supplementary Figure S1

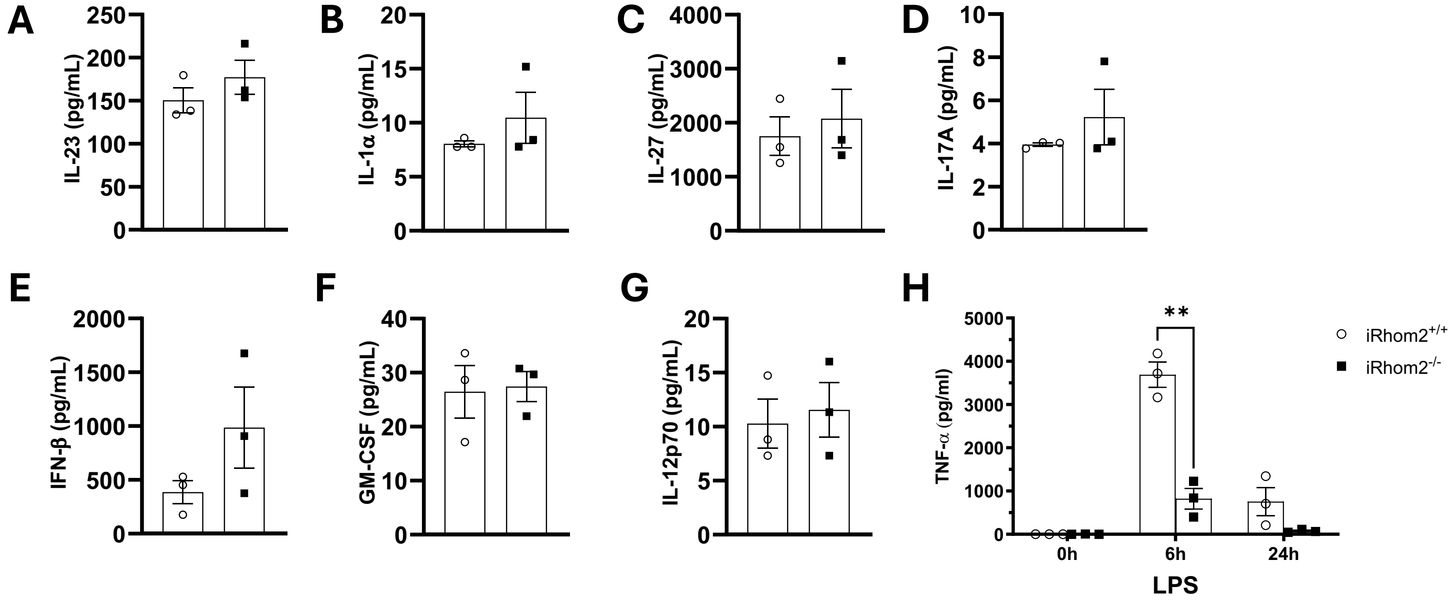

Supplementary Figure S1. Secretion of inflammatory mediators by lipopolysaccharide-stimulated bone marrow-derived macrophages.

(**A-G**) bone marrow-derived macrophages (BMDMs) from iRhom2$^{+/+}$ and iRhom2$^{-/-}$ mice were stimulated with lipopolysaccharide (LPS) for 6 h, and supernatants were analyzed using a bead-based immunoassay. Shown are levels of (**A**) interleukin-23 (IL-23); (**B**) IL-1α; (**C**) IL-27; (**D**), IL-17A; (**E**) interferion-beta (IFN-β); (**F**) granulocyte-macrophage colony-stimulating factor (GM-CSF); (**G**) IL-12p70. *n* = 3 independent experiments. (**H**) BMDMs from iRhom2$^{+/+}$ and iRhom2$^{-/-}$ mice were stimulated with vehicle control (0 h) or LPS for 6 and 24 h. TNF-α levels in the supernatant were assessed by enzyme-linked immunosorbent assay (ELISA). *n* = 3 independent experiments. **$p < 0.01$ versus genotype, determined by two-way ANOVA followed by Sidak‘s multiple-comparisons test. Data are presented as mean ± SEM with individual data points. iRhom2, inactive rhomboid protein 2.
